# Learning from patients: trainers’ use of narratives for learning and teaching

**DOI:** 10.3399/bjgpopen17X100581

**Published:** 2017-01-09

**Authors:** John R Skelton, Margaret O’Riordan, Anna Berenguera Ossȯ, Jackie Beavan, Katharine Weetman

**Affiliations:** 1 Professor of Clinical Communication, Institute of Clinical Sciences, University of Birmingham, Birmingham, UK; 2 Medical Director, Irish College of General Practitioners, Dublin, Ireland; 3 Psychologist, Institut Universitari d’Investigació en Atenció Primària, IDIAP Jordi Gol and Universitat Autònoma de Barcelona, Bellaterra (Cerdanyola del Vallès), Barcelona, Spain; 4 Freelance researcher, Interactive Studies Unit, University of Birmingham, Birmingham, UK; 5 Doctoral Researcher, Department of Primary Care Clinical Sciences Warwick Medical School, Warwick, UK

**Keywords:** doctor–patient relations, learning, physician patient relationship, primary care, professionalism, narrative medicine

## Abstract

**Background:**

There is a growing interest in how doctors learn from narratives about individual cases, reflected, for example, in the use of e-portfolios.

**Aim:**

This study aimed to evaluate how GP trainers conceptualised ‘learning from patients’, and what use they currently made of narrative recounts in training.

**Design & setting:**

Thematic analysis (TA) and corpus-linguistic (CL) analysis, with data collected from a convenience sample of trainers in the UK, Ireland, and Spain.

**Method:**

GP trainers in the three settings were contacted, and volunteers recruited (22 in UK, 24 in Ireland, and 16 in Spain). Volunteers were interviewed and asked to offer a narrative about ‘a patient you learned from’ and whether they used narratives as a training device.

**Results:**

There were no differences between settings. Trainers described an engaged and personal relationship with patients. They described learning about themselves, the human condition, and about how to live and die well. Their narratives were structured in various ways. At times, they led to precise conclusions: at times, they were perceived as meaningful, but resisting analysis. As regards teaching through narrative, it was reported as commonly used, but present practice appears ad hoc rather than planned.

**Discussion:**

The lack of difference between settings suggests a degree of commonality about how trainers perceive learning and teaching in the areas explored, but cannot be generalised further. The level of personal engagement was more than anticipated, and suggests the label ‘doctor–patient relationship’, as the term is used, may not be adequate to describe the nature of some interactions.

## How this fits in

The study of individual cases and the narratives through which they are phrased is widely used — for example, through e-portfolios — to help doctors understand the clinical and wider context of their job, and to develop professionally. The narratives trainers tell about their own learning and teaching through individual cases, however, suggests ‘learning’ is perceived often as involving wisdom about the human condition, rather than issues usually covered by such terms as doctor–patient relations.

## Introduction

The significance of narrative-based medicine has been widely discussed,^1–4^ and has influenced the development of ‘professionalism’ as a concept.^5^ Narrative, it is argued, offers ‘a possibility of understanding which cannot be arrived at by other means’,^6^ and is of value not just for patient stories, but ‘stories about being a medical professional’.^7^ Within the US at present, (echoing similar comments in for example, The Kennedy Report in the UK^8^), there is growing interest in the interactions doctors have with themselves (such as, reflection), patients, colleagues, and community,^9^ with narrative training for faculty advocated in consequence.^10^

In education generally, the point of ‘narrative recounts’ across disciplines has been defined as ‘to consider the processes of … personal and professional development’.^11^ The view of medical practice consisting of a series of ‘parables’^4^ is echoed in the rationale for the e-portfolio and the specific cases which appear in them, and also in educational theory across disciplines which advocate case-based, inductive approaches (that is, they derive general truths from the study of particular instances),^12^ in the current emphasis on the importance of critical incidents (CIs)^13^ and, traditionally, the importance of case reports in medical journals.^14^ There are some studies within medical education of the pedagogy of using narratives;^15^ within nursing, ‘narrative pedagogy’ may be better established.^16^ Finally, narratives are said to promote resilience and prevent burnout among physicians.^17^

On this basis, the doctor as ‘good professional’ is to be understood as someone capable of learning from particular events, such as those set out in a narrative, and by extension as someone capable of understanding them in all their complexity. For doctors in a teaching role this also implies the ability to facilitate the development of these attributes in others.

Therefore, this study considered how GP trainers reflect on their own experiences with patients, and use narrative accounts of such experiences for educational purposes.

’General practice’ itself changes and develops, and the details of what the phrase entails in different settings may also vary. For this reason, the present study was undertaken in three countries — Ireland (throughout the Republic), the UK (Midlands), and Spain (Barcelona) — which it was felt were in relevant areas similar in their approach to training,^18^ to explore whether there were obvious differences in what trainers discussed as part of their teaching.

## Method

This is a narrative-based evaluative study, building in part on previous work, and with a similar approach.^19^ As in the earlier study, thematic analysis^20 ^(TA) was used for broad-based qualitative study of themes, with corpus linguistics (CL)^21 ^used for detailed study of lexical patterns, particularly to seek confirmation or otherwise of tentative conclusions.

Twenty-four GP trainers were recruited in Ireland, 22 in the UK, and 16 in Spain. Trainers were approached by letter and invited to participate in a study asking them about ‘a patient you have learned from’. This formed the starting point for the interview, with interviewers encouraged simply to facilitate reflection from the trainer. There was a second question about the extent to which trainers used narratives for teaching purposes. Interviewers were asked to intervene as little as possible, since it is central to narratives that the structure and approach is the narrator’s choice. A minimal topic guide was therefore used (Appendix 1).

Interviews were conducted by a mix of GPs (trainers and non-trainers), and lay people with substantial experience in medical education. They were audiorecorded, and subsequently transcribed. Formal ethical approval was granted in Ireland and Spain. In the UK, the study was treated as an evaluation of an aspect of the existing GP training service.

A TA was undertaken, following the six ‘steps’ advocated, with the coding framework agreed by all authors, and data discussed. TA was used because it is well-validated for qualitative study, particularly, although not exclusively, in the health professions, and proved successful in the previous study.^19^ The researchers are from widely different backgrounds (two GPs, two from a language background, and one with a background in nursing and language studies): it was felt that this was sufficient precaution against prior views affecting conclusions. With three countries involved, however, much of the discussion was online.

CL has been a core methodology within language studies since the 1980s, and applied to the study of health-related texts by members of the present team and others.^22,23^ The basic methodology only is used here, designed to identify keywords, and common collocations (that is, words or phrases which frequently occur in close proximity). For present purposes, CL is used only to explore aspects of the qualitative analysis.

Spanish interviews were conducted in Spanish (*castellano*) rather than Catalan. They were translated professionally, with translations checked by two authors (who read both English and Spanish), and incorporated into the database.

## Results

‘Learning’ and ‘teaching’ inevitably appeared as the major themes. Within these, there was an additional agreement that although labels typical of medical education could be used to describe themes, many narratives stretched the meaning of such terms into new territory. In particular, ‘learning’ was based on a level of mutual personal engagement which is not covered by the term ‘doctor–patient relationship’. And what GP trainers discussed as their learning was frequently to do with what they learned about how to live and die well.

There were no meaningful differences between the three settings in how trainers conceptualised ‘learning about patients’, or about how they made use of this understanding in training, and therefore draw on all three in what follows.

### Theme 1: learning from patients

The relationship between doctor and patient was central to most narratives. However, doctors demonstrated that they perceived the distinction between professional and personal life as porous, and repeatedly breached.

#### Reciprocity

Many trainers stressed a reciprocal relationship: the need to ‘give something of your own nature’ in order to create ‘a consultation at a different level’ (I14). Thus, one GP (UK19) kept ‘personal effects on my wall or my desk’, because ‘showing some of me seems to strengthen [the] relationship’. Another (UK08) spoke of his wife’s pregnancy problems to a lady on IVF: the result of this ‘emotional resonance’ was, he was told, ‘you gave me hope’. (The patient finally conceived naturally). In conclusion, said UK08:

‘It does enhance the relationship … when you share that piece yourself’.

The result could be personal in unexpected ways. An older lady, out of the blue, told her (male) GP (S3) ‘You know doctor that I love you very much’.

#### Learning about oneself

The ‘learning’ involved was often about oneself, or the human condition. For example, I14 (quoted above) continues:

‘What I learned is that you can actually develop an awful lot of resilience from seeing what [*has*] happened … I honestly feel after dealing with him … it is possible I could have something coming up in the next 10, 15 years and I hope that having dealt with that kind of man I would be reasonably probably, I need to choose my words now, I would be reasonably confident that I would take what would come my way and I would accept it and I would accept what’s at the end of it, the fear say of say dying would have gone from me, particularly having dealt with him because he has faced it and I have faced it with him and I am reasonably ok about the fact that you know I am always testing my own reaction by seeing it happening in him and I think I would be ok.’ (I14)

Similarly, S9 spoke of her response to dying patients:

‘… we question our beliefs about death or how we ourselves fear death or how our parents died, which is a subject that always touches us’.

Another trainer told a story about missing a diagnosis early in his career: the patient came in with his son, who started ‘basically giving out to me’. But the father intervened on the young doctor’s side:

‘Our job is not black and white … he understood that through life experience … Gave the two of us a little lesson in life …’ (I18)

This trainer (S8) began to talk about working in a difficult neighbourhood:

‘… where patients have a low sociocultural level, most of them, not all — well, they often swear or use verbal aggression as a means of coercion, to make the doctor do what they want him/her to do, right? They are very suspicious, too. Well, they behave in this way until you understand their situation and learn how to handle it, and put the patient in a context, the patient across the table, right?’ (S8)

Another (S3), who talked of seeing what a ‘good death’ is, also picked up the point about learning from the adversity of others; though making the point that sometimes one learned how not to be:

‘… you learn from so many patients, from some more from some less. You learn from the way they cope with their illnesses, with the social problems of a rough area, how they survive despite the very few resources available, and yet they seem somehow happy. Having said that, you also learn from what they shouldn’t do, how some people can’t cope any more …’ (S3)

In many cases, the trainer wanted to sketch a patient who had qualities admirable in themselves, to which they too might aspire. Thus (S6) a gentleman, now of advanced age and a former international athlete, maintained an active lifestyle, and still had a ‘will to live and fight’. (His GP now ‘goes jogging’). Another narrative (S5) was of an individual during gender reassignment (male to female) who showed the (female) GP something of ‘the different ways to be a woman’. One narrative (S14) focused on a carer described as a ‘heroine’:

‘[*She gave*] loving and exquisite care, and when her mother died and then her father, or at any rate the husband of this lady, she rescued her mother’s sister … whom she hardly knew. [*A*]nd this woman seemed to me to have an extraordinary inner strength and tremendous intellectual and practical abilities. [*We think of carers as*] women, older, poor, sick, and emotional. That’s how we describe them these days, instead of saying that they are women, women, who are doing this because they are women, with the nobility of being a woman … She never spoke of economic hardship … [*s*]he was not depressed … so she didn’t fit in our stereotype … And that was also very interesting from my point of view because I think that … the medical gaze makes people’s lives look sad …’ (S14)

What precisely ‘learning about oneself’ entails could however be more complex (UK20):

‘… and the following morning I received a phone call from casualty to say that the child had been brought in dead. And so I was in the middle of my morning surgery erm I dealt with the phone call and … that was the start of a very long journey … I carried on doing my evening surgery because that’s what you do, and I think the news gradually filtered through and I spent the weekend crying pretty much really… [*Then*] I could have sent a letter but none of that actually felt right but I was very scared, I was very scared to go and see [*the mother, but I did*] … and I spent an hour … hugging this distraught, beyond distraught woman.’ (UK20)

The mother made it clear, (despite the possibility, at this stage, that the GP was to blame; she was exonerated in due course), that she wanted to continue seeing her. The GP talked about her reaction:

‘So it was a very, a very compartmentalised time almost, because you have a professional response to that patient … add to that almost not wanting to understand how she felt because you wouldn’t want to experience it in your own mind and alongside that a very personal and emotional response to having been involved in something really traumatic … going through a lot of self-reflection, self-doubt.’ (UK20)

The level of personal engagement carried over into discussions of truth-telling. The ‘right’ thing to do was — in a somewhat Aristotelian manner — seen as dependent on individuals and the situation. Honesty was generally a good thing:

‘… it gave me permission … it gave me more confidence.’ (UK21)

A lack of honesty was troubling: thus with one patient, the unspoken ‘contract’ meant GP and patient entered into a ‘dance [of denial] around the obvious’ (I14). Yet sometimes, as with a young patient dying of cervical cancer:

‘I now respect denial as a very good coping strategy for some.’ (UK11)

#### Continuity

The intersection of personal and professional life is echoed in the discussion of continuity of care. Thus (S15), ‘continuity is important … that’s what we have’. One trainer, a GP of 35 years’ standing, talked of not wanting to hand patients over to palliative care, because he ‘loved the nearness to the dying person’:

‘You build up a relationship … I don’t know what it does to me but it’s very nice to be there at the end.’ (I21)

As regards teamwork: a dying patient seemed unlikely to reach her wedding day, but the GP (UK15) and colleagues worked together, enabling the patient to get married. It was the personal, not the professional, that mattered, ‘we … rose above [the protocols]’.

### Theme 2: narratives as educational methodology

There were two issues here. One was whether trainers used narratives when teaching, and the other was how they interpreted them.

#### Teaching with narratives

This study identified what GPs volunteered about their use and understanding of narratives. In fact, though most volunteered the information that they used them, this appeared to be ad hoc, and not guided by any educational principle beyond the common sense view, as one GP(UK17) joked, that ‘general practice is a series of anecdotes, four anecdotes a day!’:

Interviewer (I): ‘*Do you ever use patient anecdotes or stories in CME or in your trainers’ workshop?’*

I12: ‘*Again inevitably. You can’t have a medical conversation without referring to these things, are you looking for an example?’*

I: ‘*No, not necessarily. It would be something that you’d think would be just quite common enough that that’d be the way you do it.’*

I12: ‘*Well of course, “I saw this patient yesterday”, isn’t that how a lot of these stories start? “I had this patient in last week”, isn’t that how an awful lot of it starts?’*

Thus, for example, teaching through narrative was common, but ‘not formally programmed’:

I10: ‘*… I’ve discussed [stories] with people in the practice yeah.’*

I: ‘*And would that be as a learning tool directly?’*

I10: ‘*No, I haven’t formalised it into a learning tool but maybe that’s something that we could think about doing.’*

Similarly, despite the common practice of using case analysis in GP training, this was mentioned only once (by S9) as an example of narrative, although it is. In addition, there were mentions of narratives being (appropriately) put onto social media. However, no trainers volunteered an educational rationale for narrative-based teaching. One, however, mentioned the risk that narratives might be mistaken for evidence:

‘I’m very wary of anecdotes, “Oh I tried this drug it worked for so and so …” Therefore: I wouldn’t bring all these stories [*into training*] because I think most doctors have the same stories”.’ (I12)

#### Ascribing meaning

Some narrative types are designed to point to a simple conclusion. This is true of parables (’Don’t pass by on the other side’), fables (’Don’t cry wolf’), or whodunnits (’The butler did it’). Others are designed to resist reductionism (The ‘meaning of *Hamlet’* is not ‘Don’t procrastinate’). The range was represented in the data.

Thus, one GP had jumped to the conclusion that a patient’s problem was alcohol-induced, because of her history: but it was not. Therefore:

‘Don’t blame … results on … past history.’ (UK4)

Similarly, a patient who walked into surgery turned out to have a fractured spine:

‘I learned … just because somebody walks in … maybe I should be quicker to refer for X-ray.’ (I11)

And I4, reflecting on how a patient with multiple sclerosis ‘organise[s] his own life’, concluded:

‘… our support may not be the support we always think we need to give. Maybe it’s the support they decide that they want us to give.’ (I4)

The more profound the narrative, however, the less reducible the conclusion. I4, who misdiagnosed a patient, offered a summary echoing the theme of reciprocal engagement:

“… patients rely on their doctors but the doctors rely on the patients, I suppose that’s the lesson … as well’ [*that is, as well as other lessons*].’ (I4)

Similarly a patient, over many years, received three separate diagnoses of cancer. What impressed the GP (I1) was his ‘acceptance’:

‘… you came away enriched by the whole thing.’ (I1)

But what exactly was ‘the whole thing’? As the GP talked, it became clear that the ‘moral’ was — irreducibly — the whole person:

‘So that was one standout experience in the last 20 years that I’ve had. I’ve had a couple of other similar ones but none that capture the whole picture of total patient care and someone’s complete philosophy of life and somebody’s acceptance of incredible adversity and he was then obviously so caring and supporting of someone else close to him who was ill [*that is, his partner*] even though he was very ill.’ (I1)

UK20, talking of the child who died, and seeking to identify meaning, offered at least six conclusions (The first is cited as a quotation, although it is not exact):

1. *“If you ask for courage you might be given an opportunity to show courage”.*
2. *Living with those mistakes is what is actually a part of who you are.*
3. *There’s a power imbalance that we’re not aware of as clinicians.*
4. *The easy thing … would be to hide behind that barricade of professionalism … The hardest thing for me was to step from beyond that and make myself personally vulnerable.*
5. [We had to] *make a way forward which was all about relationship, not about science or fact.*
6. *There is something for registrars to realise that however bad it is, it’s not that bad.* (UK20)

Sometimes no attempt is made to summarise: narratives speak for themselves. First, an apparently trivial moment. A mother came with her (usually very serious) son, who had learning difficulties. The GP had a medical student with him:

‘Suddenly the patient started to laugh, first to smile, then to laugh and we were a bit surprised, never seen him doing that before, usually he has some kind of suffering: mental or physical perhaps. The mother said it’s because of the student, she’s got such a lovely smile. This was serious; it was not in jest really. And we found ourselves laughing our heads off, the four of us. It was quite moving.’ (UK18)

Finally, a GP talked of a lady in early middle age who had been very ill:

‘… I suppose I was fortunate enough that when she actually died I was on call that night, it was when I used to cover the [*out of hours service*], which I don’t tend to work at night anymore, but I did then and er got called to certify the death, which we don’t do anymore, it’s usually paramedics but then I did go out and I was just amazed that when I walked in, they had, the family were all there, I’m getting choked up! But the whole family was there, and they’d put those little candles, you know the tea lights, they’d put them all up the stairs to her bedroom …’ (UK16)

This point of the narrative is to lead to this single irreducible and profound image of the doctor’s role as a witness to love and respect.

## Language analysis

At the basic level of language analysis appropriate for this study, it was felt reasonable to treat a translation as if it was an original. The total database of words spoken by GPs comprised 120 295 words.

Language analysis was used subsequent to qualitative analysis, in particular to explore details of findings for the balance between the professional and the personal.

Results confirmed the qualitative analysis. A list of the most common nouns is shown in Table 1. It will be seen that, though ‘doctor’, ‘GP’ and ‘patient’ are — inevitably — common, many of the words cluster around personalised concepts: ‘person/people’, ‘life’, ‘experience’ and so on. (Note, too, the high frequency of ‘cancer’, linked in the narratives with the idea of learning how to die) .

**Table 1.** Common nouns in all data

Rank	Occurrences	Noun
1	795	patient/patients
2	625	thing/things
3	495	people/person
4	433	time
5	393	years/year
(=)6	231	way
(=)6	231	doctors
7	164	family
8	156	practice
9	141	life
10	130	day
(=)11	125	case
(=)11	125	experience
12	114	cancer
13	114	home
14	112	work
15	110	go
16	100	relationship
17	90	hospital
18	86	months
19	89	problem
20	86	situation
21	82	lady
22	80	consultation
23	79	job
(=)24	77	fact
(=)24	77	point
25	67	illness

The phrase ‘doctor–patient relationship’ occurred just four times, and the phrase ‘continuity of care’ twice. However, the term ‘relationship’, not collocated with ‘doctor–patient’, occurred 96 times. Clearly, this allowed for a degree of ambivalence: individual GPs may often be unsure whether they have the personal or the professional in mind. But it was noticeable that ‘relationship’ frequently collocated with evaluative terms such as ‘fairly’ ’very’, good’, ‘strong’ (Box 1), which have a sense of psychological rapport. More overtly professional evaluations were absent: no-one said their relationship was ‘therapeutic’, or ‘appropriate’, for example. Similarly, the word ‘personal’ occurred 44 times.

**Box 1.** Examples of evaluative use of ‘relationship’, all data.

this was diagnosed and we had a fairly good	relationship	She trusted me. If I came across any
at the time cos we had such a good	relationship	And it’s all for the good of their
and all that so we had a very good	relationship	So there’s another part of that. D:
go backwards and forwards and had quite a strong	relationship	With her and I could have sent a lett

## Discussion

### Summary

This study explored what GP trainers mean when they say they ‘learn’ from patients, as a means of understanding present educational practice.

‘Learning’ was at a personal level. There was a clear emphasis on the doctor and patient as people rather than (merely) occupants of social or professional roles. There was evidence of trainers learning how to live and die well; a strongly engaged stance to the ethics of truth telling; and a willingness to ‘give themselves’. The sense of human engagement entails an overall involvement, sustained over a period of time, with the life of the patient and those around them. This involvement includes a knowledge of all those involved as people, and existing in a community of which the GP is also a member.

The narrative methodology created opportunities for the kind of deeper engagement that is on display, but there are difficulties with narratives as a teaching device. They are particular stories which may exemplify general truths, or may be told precisely because they are unusual, and because only narrative complexity rather than summary statements does justice to the event. Narrative methodology was almost never something that trainers discussed.

### Strengths and limitations

Data were supplied by 62 trainers in three countries. It was not possible to identify differences between settings. This suggests a degree of commonality: but the doctors were volunteers, so representative only of that group.

The main strength of the study was the unexpected way in which relationships are described. The degree of personal engagement is not well captured by standard use of such terms as ‘doctor–patient relationship’.

The authors are reasonably confident that no cultural nuances have been missed in the data, but no translation is a perfect mirror. They did not specifically enquire about links with critical incidents (CIs) and significant event audit (SEAs), nor with case analysis: nor more generally did they introduce methodological issues in interview. They wanted the emphasis of the discussion to be clearly on the story telling. This may inadvertently have made it harder for trainers to articulate a more sophisticated sense of the function of narratives.

### Comparison with existing literature

The tradition of thought and research into the ‘doctor–patient relationship’ goes back decades. What this study found was more akin to Balint’s concept of ‘mutual investment’,^24^ than more modern formulations of ‘partnership’,^25^ in that data suggest a psychological commitment from the GP. The educational principles informing narrative, inductive learning,^12^ or the value of ‘bearing witness’,^26^ were not mentioned, but the authors did not ask explicitly about theoretical understanding.

In a systematic review of reflection,^27^ Mann and colleagues mention ‘three definitions’. One is from Dewey^28^ (as Mann and colleagues say, offering a version of ‘critical thinking’). One is from Moon,^29^ who talks of ‘mental processing … applied to relatively complex or unstructured ideas’, and also of the ‘emotional’ aspect of reflection: and one is from Boud,^30^ who focuses on ‘intellectual and affective activities in which individuals engage … in order to lead to a new understanding and appreciation’. The concept of complexity is important: so too is the idea of an affective element, and clearly many of these narratives had a powerful emotional impact on the doctors concerned. However, this study draws attention to the fact that narratives — from a doctor, or from a novel — allow us to make sense of a complex world which we cannot (returning to Greenhalgh’s point^6^) understand by other means.

### Implications for research and practice

The ad hoc use of narratives in teaching was unsurprising: and if anything responders may have politely overstated their use. Equally unsurprising was the fact that GPs spoke little about the educational rationale for using, or avoiding, narratives: as is appropriate with TA, the authors looked hard for evidence to the contrary, but found little. A study targeted exclusively on this issue might be of value. Given the depth of the stories in the data, this study advocates a more confident reliance on them (although not as if they were research evidence), and a better understanding of how narratives resolve or sustain complexity. This argues for better training in the educational principles of using narratives.

One simple reason for using narrative is that the stories which were told cannot but help individual GPs, trainers, and registrars alike, to think positively about the profession, and about their own lives.

## Appendix 1 Topic guide

### Structure for Interview

1. Sign consent form
2. Demographics
  *What year did you qualify from medical school?*
  *Which medical school was it?*
  *How long have you been a GP?*
  *How long have you been a GP Trainer?*
3. Main questions
  - Can you tell me about a patient that you have learnt something from?
    Supplemented if necessary:
      - *Can you describe an encounter you had with them?*
      - *Was it a one-off encounter or a series of encounters (at what stage of career as a GP did the encounter happen — will probably come out naturally in the story but might need to be asked)*
      - *Did it change the way you worked?*
  - Have you shared this learning experience with other doctors — peers, trainers, medical students, GP trainees? Can you describe this?
